# Comparison Between Diagnostic Nasal Endoscopy and Plain Computed Tomography of the Paranasal Sinuses in Chronic Rhinosinusitis

**DOI:** 10.7759/cureus.112978

**Published:** 2026-07-19

**Authors:** Manognya Ryali, G K Narayana, Adharsh AD

**Affiliations:** 1 Otolaryngology - Head and Neck Surgery, Sri Devaraj Urs Academy of Higher Education and Research, Kolar, IND; 2 Radiodiagnosis, Sri Devaraj Urs Academy of Higher Education and Research, Kolar, IND

**Keywords:** chronic rhino sinusitis, ct-pns, deviated nasal septum (dns), diagnostic nasal endoscopy, functional endoscopic sinus surgery (fess), osteomeatal complex

## Abstract

Background

Chronic rhinosinusitis (CRS) is a persistent inflammatory condition of the nasal cavity and paranasal sinuses lasting more than 12 weeks despite appropriate medical treatment. Its symptoms include nasal congestion, nasal drainage, facial pain, and loss of smell. The diagnosis of rhinosinusitis is essential to managing the condition effectively. Endoscopy and CT of the paranasal sinuses (CT-PNS) are two main imaging methods used to diagnose the condition. Endoscopy enables direct visualization of the nasal cavity, whereas CT provides detailed imaging of the paranasal sinuses. Neither imaging method is sufficient on its own, but each has advantages and disadvantages for the diagnostic process. Understanding these differences is essential to establishing an optimal diagnostic procedure for patients with CRS.

Materials and methods

This is a cross-sectional observational study conducted in patients with CRS in the department of otorhinolaryngology OPD with a sample size of 70. Patients were selected based on inclusion and exclusion criteria. The procedure was explained, and informed consent was obtained. They underwent diagnostic nasal endoscopy (DNE) and plain CT-PNS, and the findings were documented for comparison.

Results

CT positivity (88.6%) was higher than DNE positivity (82.9%), and dual positivity was observed in 77.1% of patients. The diagnostic validity of DNE showed a sensitivity of 87.1%, a specificity of 50.0%, a positive predictive value of 93.1%, and an accuracy of 82.9%. There was a statistically significant association between modalities, though the correlation between severity scores was weak and not significant.

Conclusions

DNE and CT-PNS are used to assess CRS, although they are complementary. DNE is highly effective as an initial, office-based diagnostic modality with good sensitivity and predictive value. In contrast, CT-PNS is necessary for overall evaluation, assessment of posterior sinus involvement, and operative planning.

## Introduction

Chronic rhinosinusitis (CRS) is a persistent inflammatory condition of the nasal passages and paranasal sinuses, defined by the presence of at least two cardinal symptoms: nasal obstruction, nasal discharge, facial pain or pressure, and hyposmia or anosmia, lasting twelve weeks or more despite appropriate medical therapy [1]. The condition affects a significant proportion of the global population. It imposes a substantial burden on healthcare systems, with its impact on quality of life comparable to that of other chronic diseases such as diabetes mellitus and chronic obstructive pulmonary disease. Given the overlapping symptomatology of CRS with allergic rhinitis, vasomotor rhinitis, and upper respiratory infections, accurate diagnosis requires objective confirmation using validated diagnostic modalities rather than clinical history alone [1,2].

Diagnostic nasal endoscopy (DNE) is a cornerstone in the evaluation of CRS, offering direct, real-time visualization of the nasal cavity and key anatomical landmarks, including the middle meatus, osteomeatal complex, and sphenoethmoidal recess. Performed in an outpatient setting with minimal discomfort, DNE enables identification of mucosal edema, nasal polyps, purulent discharge, crusting, and anatomical variants such as septal deviations and concha bullosa. The Lund-Kennedy endoscopic scoring system provides a standardized, reproducible method for grading disease severity based on these findings. It has been widely adopted in both clinical practice and research settings [2,3]. Furthermore, DNE facilitates targeted microbial culture sampling and tissue biopsy and serves as an invaluable tool for postoperative surveillance and longitudinal monitoring of treatment response.

CT of the paranasal sinuses (CT-PNS), particularly non-contrast thin-section imaging in axial and coronal planes, is regarded as the radiological gold standard for assessing sinonasal anatomy and disease extent in CRS. CT provides detailed cross-sectional visualization of bony architecture, mucosal thickening, sinus opacification, osteomeatal complex obstruction, and anatomical variants, including Haller cells, agger nasi cells, and Onodi cells, which may predispose to impaired sinus drainage and recurrent disease. The Lund-Mackay scoring system allows objective quantification of sinus opacification. It is widely endorsed by clinical guidelines, including the European Position Paper on Rhinosinusitis and Nasal Polyps (EPOS) and the American Academy of Otolaryngology-Head and Neck Surgery (AAO-HNS) [3,4]. However, CT findings do not invariably correlate with symptom severity or active mucosal inflammation, and incidental radiological abnormalities may be encountered in asymptomatic individuals, underscoring the need for clinical correlation.

While both modalities are integral to CRS evaluation, each possesses inherent strengths and limitations. DNE excels at detecting active mucosal inflammation and anterior sinus pathology but is limited in visualizing deep-seated or posterior sinus disease. Conversely, CT offers a comprehensive, operator-independent assessment of all paranasal sinuses and is indispensable for preoperative planning. However, it provides only a static depiction and cannot assess real-time mucosal dynamics. Several studies have reported moderate-to-strong concordance between endoscopic and CT findings, particularly for maxillary and anterior ethmoid disease, with discrepancies noted in frontal and sphenoid sinus involvement [4-6]. The present study was therefore undertaken to compare findings between DNE and plain CT-PNS in patients with CRS to evaluate the diagnostic agreement, sensitivity, specificity, and complementary value of these two modalities in a tertiary care setting.

## Materials and methods

Study design and setting

This was a cross-sectional observational study conducted in the Department of Otorhinolaryngology, Sri Devaraj Urs Medical College, Tamaka, Kolar, Karnataka, India, over an 18-month period (April 2024 to October 2025). The study protocol was approved by the Central Ethics Committee of Sri Devaraj Urs Academy of Higher Education and Research (approval number: SDUAHER/KLR/R&D/CEC/S/PG/56/2024-25), and written informed consent was obtained from all participants prior to enrollment.

Study population

Patients presenting to the ENT outpatient department with clinical features suggestive of CRS were screened for eligibility. A total of 70 patients who fulfilled the inclusion criteria were enrolled using a convenience sampling method.

Inclusion and exclusion criteria

Patients aged 18-65 years presenting with at least two major symptoms of CRS (nasal obstruction, nasal discharge, facial pain or fullness, or reduced sense of smell) persisting for ≥12 weeks, in accordance with the European Position Paper on Rhinosinusitis and Nasal Polyps (EPOS 2020) diagnostic criteria [7] and with mucopurulent discharge observed in the middle meatus during preliminary examination, were included in the study.

Patients with polypoidal lesions or expansive nasal masses, nasal deformities or prior trauma to the nasosinusal region, facial deformities affecting sinus anatomy, and symptoms unrelated to CRS (e.g., acute sinusitis or neoplasms) were excluded from the study.

Sample size estimation

The sample size was estimated using the method described by Buderer for diagnostic accuracy studies, which incorporates the anticipated sensitivity of the index test and the estimated disease prevalence. The number of disease-positive subjects required to estimate sensitivity was calculated as

$n = \frac{Z_{1-\alpha/2}^{2}\,\mathrm{Se}\,(1-\mathrm{Se})}{d^{2}}$

and the total sample size as

$N = \frac{n}{P}$

where Z₍₁₋α/₂₎ = 1.96 corresponds to a 95% confidence level, Se is the expected sensitivity of DNE, d is the absolute precision, and P is the expected prevalence of CT-positive disease.

Assuming an expected DNE sensitivity of 84.61% with an absolute precision of 10%, the required number of disease-positive subjects was approximately 50; applying an expected CT-positive prevalence of 71%, the minimum total sample size was 70. The reported CT sensitivity of 99% yields a substantially smaller requirement and was therefore not the binding constraint. Accordingly, 70 patients fulfilling the inclusion criteria were enrolled in the study.

Study procedure

All enrolled participants underwent a detailed clinical evaluation including history-taking and anterior and posterior rhinoscopy. DNE was performed under topical anesthesia (4% lidocaine) using a 30° rigid endoscope. Three systematic passes were made to examine the inferior meatus, sphenoethmoidal recess, and middle meatus. Endoscopic findings were scored using the Lund-Kennedy [8] scoring system, which grades polyps (0-2), mucosal edema (0-2), discharge (0-2), scarring (0-2), and crusting (0-2) for each nasal cavity, yielding a maximum score of 20. DNE positivity was defined as a Lund-Kennedy score >2.

Subsequently, all patients underwent non-contrast CT-PNS in the axial and coronal planes with 2-mm resolution. CT findings were scored using the Lund-Mackay scoring system [9], which grades opacification (0 = none, 1 = partial, 2 = complete) for each of five sinus groups bilaterally and osteomeatal complex obstruction (0 = not obstructed, 2 = obstructed), yielding a maximum score of 24. CT positivity was defined as a Lund-Mackay score >4. Anatomical variants were also documented.

Statistical analysis

Data were entered into Microsoft Excel (Microsoft Corp., Redmond, WA, USA) and analyzed using OpenEpi software (OpenEpi: Open Source Epidemiologic Statistics for Public Health, Atlanta, Georgia, USA. Available at: https://www.openepi.com). Descriptive statistics (mean, standard deviation, frequencies, and percentages) were used to summarize demographic and clinical variables. The association between DNE and CT positivity was assessed using Pearson's chi-square test. Diagnostic validity of DNE against CT (as the reference standard) was evaluated by computing sensitivity, specificity, positive predictive value (PPV), negative predictive value (NPV), and overall accuracy. Agreement between the two modalities was assessed using Cohen's kappa coefficient. Correlation between Lund-Kennedy and Lund-Mackay scores was evaluated using Spearman's rank correlation coefficient. The association between symptom duration and severity scores was tested using one-way ANOVA. A p-value of <0.05 was considered statistically significant.

## Results

A total of 70 patients with clinically diagnosed CRS were enrolled. The mean age was 43.50 ± 10.69 years (range: 23-69 years), with the majority belonging to the 41-50 years age group (34.3%). Females constituted 54.3% (n = 38) and males 45.7% (n = 32). The most frequent presenting symptoms were nasal obstruction (90.0%) and nasal discharge (85.7%), followed by facial pain or pressure (67.1%) and decreased sense of smell (51.4%). Bilateral disease (64.3%) was more common than unilateral involvement (35.7%). Allergic rhinitis was the most prevalent comorbidity (35.7%), followed by asthma (14.3%).

On DNE, mucosal edema was the most common finding (82.9%), followed by middle meatal secretions (78.6%), inferior turbinate hypertrophy (47.1%), septal deviation (42.9%), and nasal polyps (21.4%). On CT-PNS, maxillary sinus involvement (78.6%) and ethmoid sinus involvement (62.9%) were most frequent, with osteomeatal complex obstruction present in 60.0% of cases. Frontal and sphenoid sinus involvement were comparatively less frequent at 28.6% and 22.9%, respectively. The most common anatomical variant on CT was deviated nasal septum (38.6%), followed by agger nasi cells (27.1%) and concha bullosa (18.6%).

The mean Lund-Kennedy endoscopy score was 5.33 ± 2.27 (median 6, IQR 2), and the mean Lund-Mackay CT score was 8.27 ± 3.56 (median 8, IQR 6), indicating a moderate disease burden on both modalities. The cross-tabulation of DNE positivity and CT positivity is presented in Table 1.

**Table 1:** Comparison of DNE positivity with CT positivity (N = 70) DNE: diagnostic nasal endoscopy, CT: computed tomography

	CT negative	CT positive	Total
DNE negative	4	8	12
DNE positive	4	54	58
Total	8	62	70

CT positivity (Lund-Mackay >4) was present in 88.6% (62/70) and DNE positivity (Lund-Kennedy >2) in 82.9% (58/70) of patients. Dual positivity was observed in 77.1% (54/70), while 5.7% (4/70) were negative on both modalities. CT-positive but DNE-negative discordance was noted in 11.4% (8/70) of cases and DNE-positive but CT-negative discordance in 5.7% (4/70). The association between DNE and CT positivity was statistically significant (χ² = 6.865, p = 0.009). The diagnostic validity of DNE against CT as the reference standard is summarized in Table 2.

**Table 2:** Diagnostic validity of DNE compared to CT as the reference standard (N = 70) DNE: diagnostic nasal endoscopy, CT: computed tomography

Measure	Value
True positive (TP)	54
False positive (FP)	4
False negative (FN)	8
True negative (TN)	4
Sensitivity	87.10%
Specificity	50.00%
Positive predictive value (PPV)	93.10%
Negative predictive value (NPV)	33.30%
Overall accuracy	82.90%

Using CT positivity as the reference standard, DNE demonstrated high sensitivity (87.1%) and high PPV (93.1%), indicating that a positive endoscopic finding reliably corresponds with CT-confirmed disease. Specificity was moderate (50.0%), and NPV was low (33.3%), reflecting that a negative DNE does not reliably exclude radiologically evident CRS. The overall diagnostic accuracy of DNE was 82.9%. The agreement and correlation between the DNE and CT-PNS scoring systems are presented in Table 3.

**Table 3:** Agreement and correlation between DNE and CT-PNS scoring systems (N = 70) DNE: diagnostic nasal endoscopy, CT-PNS: computed tomography scanning of the paranasal sinuses

Measure	Value	p-value
Cohen's kappa (DNE vs CT positivity)	0.305	0.009
Spearman's ρ (Lund–Kennedy vs Lund-Mackay score)	0.207	0.086

Agreement between DNE positivity and CT positivity was fair (κ = 0.305, p = 0.009). The correlation between the continuous severity scores, Lund-Kennedy (endoscopy) and Lund-Mackay (CT), was positive but weak and did not reach statistical significance (ρ = 0.207, p = 0.086), suggesting that while the two modalities tend to concur on the presence or absence of disease, they do not necessarily grade severity in parallel. The distribution of CT-PNS findings across the individual sinus groups and the osteomeatal complex is detailed in Table 4.

**Table 4:** Distribution of CT-PNS findings: sinus involvement and osteomeatal complex obstruction (N = 70) CT-PNS: computed tomography scanning of the paranasal sinuses

CT finding	Present, n (%)
Maxillary sinus involvement	55 (78.6)
Ethmoid sinus involvement	44 (62.9)
Frontal sinus involvement	20 (28.6)
Sphenoid sinus involvement	16 (22.9)
Osteomeatal complex obstruction	42 (60.0)

CT-PNS revealed a predominantly maxillo-ethmoidal disease pattern. The maxillary sinus was the most commonly involved (78.6%), followed by the ethmoid sinuses (62.9%). Osteomeatal complex obstruction was present in 60.0% of patients, underscoring the central role of impaired drainage pathways in the pathophysiology of CRS. Frontal (28.6%) and sphenoid (22.9%) sinus involvement were less frequent, and these posterior or superior sinuses represent the regions where CT provides the greatest diagnostic advantage over endoscopy.

## Discussion

The present study compared DNE with CT-PNS in 70 patients with CRS, evaluating their concordance, diagnostic validity, and complementary roles using standardized scoring systems. The cohort was predominantly middle-aged (mean age 43.50 ± 10.69 years), with a slight female preponderance (54.3%) and a clinical profile dominated by nasal obstruction (90.0%) and nasal discharge (85.7%). Bilateral disease was observed in 64.3% of cases, and allergic rhinitis was the most prevalent comorbidity (35.7%). These demographic and clinical characteristics are consistent with adult CRS cohorts reported by Liang et al. [1] (n = 120), Bhattacharyya [2] (n = 156), and Nathan [3] (n = 150), confirming that the study population is representative of the typical CRS burden encountered in otolaryngological practice. The predominance of symptoms in the productive adult age group reinforces the need for accurate, objective diagnostic strategies to guide timely and appropriate management.

DNE demonstrated high positivity (82.9%), with mucosal edema (82.9%) and middle meatal secretions (78.6%) being the most frequently identified findings. In comparison, CT-PNS showed a marginally higher positivity rate (88.6%) with a predominantly maxillo-ethmoidal pattern of involvement: maxillary sinus (78.6%), ethmoid sinus (62.9%), and osteomeatal complex obstruction (60.0%). The high detection rate of anterior sinus and middle meatal pathology on endoscopy aligns with the observations of Hussein and Jaf [4], who reported mucosal edema in 72% and purulent discharge in 58% of their CRS cohort. However, frontal (28.6%) and sphenoid (22.9%) sinus involvement were less frequently detected and were primarily identified on CT, consistent with the findings of Lohiya et al. [5] and Bagewadi et al. [6], who reported that endoscopy had reduced sensitivity for posterior and deep-seated sinus disease owing to anatomical limitations of visualization. CT also identified clinically relevant anatomical variants, including deviated nasal septum (38.6%), agger nasi cells (27.1%), and concha bullosa (18.6%), reinforcing its established superiority in structural mapping and preoperative anatomical delineation, as highlighted by Sriprakash and Sisodia [10] and Shahizon et al. [11].

The diagnostic validity analysis revealed that DNE, when measured against CT positivity as the reference standard, had a sensitivity of 87.1%, specificity of 50.0%, PPV of 93.1%, NPV of 33.3%, and overall accuracy of 82.9%. The high sensitivity and PPV indicate that a positive endoscopic finding reliably corresponds with CT-confirmed disease, making DNE a robust first-line confirmatory tool for CRS. These figures are comparable to those reported by Krishniya et al. [12] (sensitivity 84%, specificity 76%), Raman et al. [13] (sensitivity 85%, specificity 78%), and Sharma et al. [14] (sensitivity 85%, specificity 78%, PPV 82%, NPV 81%). The relatively low specificity (50.0%) and NPV (33.3%) in the present study can be attributed to the small CT-negative subgroup (n = 8), which makes these estimates sensitive to minor variations in counts. Notably, 11.4% of cases were CT-positive but DNE-negative, indicating that a negative endoscopic examination cannot reliably exclude CRS, particularly when disease is confined to deeper sinuses inaccessible to endoscopic visualization. This observation is consistent with Uwaneme et al. [15], who reported 18% discordance between the two modalities, predominantly due to missed posterior sinus pathology on endoscopy. The overall diagnostic accuracy of 82.9% in the present study closely mirrors the 82% accuracy reported by Kim et al. [16], further supporting the validity of DNE as a dependable diagnostic modality.

Agreement analysis between the two modalities revealed a fair level of concordance (Cohen's kappa = 0.305, p = 0.009). At the same time, the correlation between continuous severity scores (Lund-Kennedy and Lund-Mackay) was positive but weak and not statistically significant (Spearman's ρ = 0.207, p = 0.086). This indicates that although DNE and CT tend to agree on the categorical presence or absence of disease, they do not necessarily grade severity in a parallel fashion. Higher levels of agreement have been reported in comparable studies: Kumar et al. reported a kappa of 0.68, and Sheikh et al. [17] reported a correlation coefficient of 0.79 (p < 0.01) between endoscopic and CT scores. The weaker agreement observed in the present study may be explained by the high overall CT positivity rate (88.6%), which may have created a ceiling effect. Additional contributing factors include the different cut-off thresholds used (Lund-Kennedy >2 vs. Lund-Mackay >4) and the inherent differences between what each modality measures: endoscopy captures visible mucosal inflammatory activity. At the same time, CT quantifies the anatomical extent of sinus opacification, a distinction underscored by Bhattacharyya [2], who noted that symptom improvement following medical therapy does not always correlate with radiological resolution. Additionally, symptom duration showed no significant association with either endoscopic severity (ANOVA F = 1.317, p = 0.276) or CT severity (F = 1.280, p = 0.289), suggesting that chronicity alone is a poor surrogate for objective disease burden and reinforcing the necessity of formal scoring-based assessment irrespective of symptom duration.

This study supports the complementary nature of DNE and CT-PNS in evaluating CRS. DNE serves as an effective, accessible, and radiation-free first-line objective diagnostic tool with high sensitivity and PPV, particularly for anterior sinus and middle meatal disease. However, its limitations in detecting posterior and deep-seated sinus pathology, as well as its moderate specificity and low NPV, necessitate the judicious use of CT-PNS in cases of refractory symptoms, discordant clinical-endoscopic findings, suspected posterior or frontal sinus involvement, and surgical planning. A stepwise diagnostic algorithm, commencing with clinical evaluation and DNE and reserving CT for complex, recurrent, or surgically indicated cases, appears to be the most clinically rational and cost-effective approach, as also advocated by Sheikh et al. [17] and Handique et al. [18]. The integration of both modalities, supported by standardized scoring systems, provides the most accurate and clinically meaningful framework for CRS diagnosis, severity assessment, and treatment planning.

Limitations

This study has several limitations. It was conducted at a single tertiary care center using a relatively small cohort of 70 patients recruited by convenience sampling, which may introduce selection bias and limit the generalizability of the findings to broader populations. The cross-sectional design precluded assessment of treatment response, disease progression, and long-term follow-up. CT-PNS was treated as the reference standard despite the absence of histopathological or microbiological confirmation, and the small CT-negative subgroup (n = 8) rendered the estimates of specificity and NPV imprecise and sensitive to minor variations in cell counts. Endoscopic and radiological scoring may also be subject to interobserver variability, and the documented anatomical variants were not correlated with disease severity or surgical outcomes. Larger, multicentric, prospective studies with longitudinal follow-up are therefore warranted to validate these observations.

## Conclusions

DNE and CT-PNS are complementary diagnostic modalities for evaluating CRS. DNE demonstrated high sensitivity and PPV, making it a reliable, cost-effective, and radiation-free first-line investigation for detecting mucosal disease. In contrast, CT-PNS provided comprehensive information on the extent of disease, posterior sinus involvement, anatomical variations, and preoperative planning. A stepwise approach using DNE for initial evaluation and reserving CT-PNS for selected cases with persistent symptoms, inconclusive endoscopic findings, or surgical consideration offers the most accurate and clinically effective strategy for diagnosing and managing CRS.
